# Synergistic toughening of polypropylene with ultra-high molecular weight polyethylene and elastomer-olefin block copolymers †

**DOI:** 10.1039/c9ra01073d

**Published:** 2019-08-02

**Authors:** Lucheng Qi, Lei Wu, Ren He, Hui Cheng, Boping Liu, Xuelian He

**Affiliations:** Shanghai Key Laboratory of Multiphase Material Chemical Engineering, East China University of Science and Technology Shanghai 200237 China hexl@ecust.edu.cn lcqi_1224@foxmail.com edwood_vip@163.com huihyva@ecust.edu.cn +86-21-64253364; College of Materials and Energy, South China Agricultural University Guangzhou 510642 China boping@scau.edu.cn; Daqing Petrochemical Research Center of PetroChina Daqing 163714 China renhe459@petrochina.com.cn

## Abstract

Blends of polypropylene (PP) and ultra-high molecular weight polyethylene (UHMWPE) with elastomer-olefin block copolymers (OBC) were prepared using an ultrasonic twin-screw extruder, and the mechanical, thermal, and rheological properties of the blends were investigated. The interfacial interactions among PP, OBC, and UHMWPE showed that the PP/OBC/UHMWPE blends formed a core–shell structure with UHMWPE as the core and OBC as the shell. The crystallization temperature and the crystallinity of the blends were improved for the heterogeneous nucleation between PP- and OBC-covered UHMWPE particles. Moreover, the mechanical and thermal properties of PP/UHMWPE blends have also been greatly improved by adding OBC. Furthermore, it was evident that the OBC-covered HHMWPE particles became smaller under the application of ultrasonic irradiation, so the interfacial interactions between the particles and the PP matrix were enhanced and the impact strength of the blends was improved.

## Abstract 2

Blends of polypropylene (PP) and ultra-high molecular weight polyethylene (UHMWPE) with elastomer-olefin block copolymers (OBC) were prepared using an ultrasonic twin-screw extruder, and their mechanical and rheological properties were investigated.

## Graphical abstract

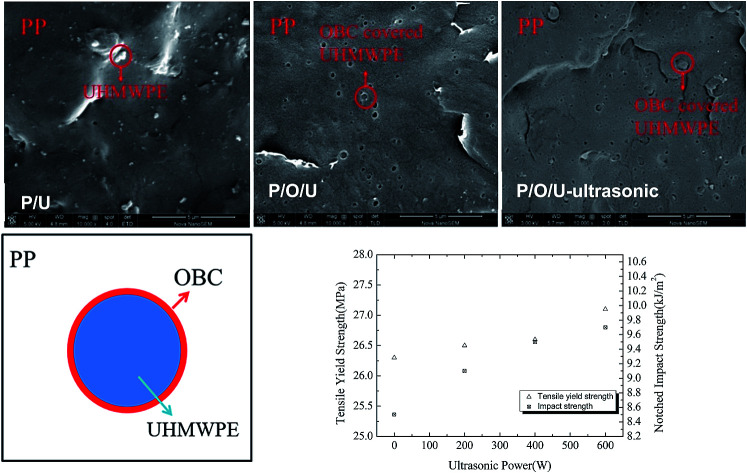

## 1. Introduction

As one of the most widely used thermoplastic plastics in the world, polypropylene (PP) has been used more and more widely in all walks of life. Research studies on high toughness polypropylene blends have great scientific significance.^1–6^ Plastic toughening polypropylene is a common method, which mainly uses high toughness polymers (HDPE, UHMWPE, *etc.*) to blend with low toughness polypropylene to prepare blends, in order to improve the impact resistance of polypropylene. However, due to the thermal incompatibility between polypropylene and toughened plastics, the properties of the blends are often unsatisfactory.^7^

Melt blending is one of the most common methods for processing polymer materials. When the two materials are dispersed unevenly due to the difference of viscosity, the screw speed is often increased to improve the dispersion.^8^ But for some polymers such as PP, if the screw speed is too high and the shear force is too large, the material will degrade and the mechanical properties will decline. The application of ultrasound in a screw extruder can solve this problem very well. The cavitation of ultrasound can produce huge energy, making the polymer disperse evenly, and the action time is short, so it will not cause serious degradation of polymer molecular chains.^9^ Feng *et al.*^10^ installed an ultrasonic device at the head of twin-screw extruder and blended melting PP and EPDM, and obviously improved the toughness of the blends. Isayev *et al.*^11^ found that carbon nanotubes dispersed well in polyetherimide (PEI) with multi-walled carbon nanotube (MWNT) content of 10 wt% when melt extruding PEI and MWNTs using ultrasonic screw extruder. Chen *et al.*^12^ studied the effect of different ultrasonic power on the properties of PP/EPDM blends and found that with the increase of ultrasonic power, the particle size of rubber phase decreased gradually.

Since the random copolymer ethylene-octene copolymer (POE) of thermoplastic elastomer ethylene-octene was developed by Dow Chemistry in the United States, polypropylene toughened using thermoplastic elastomers has gradually replaced the traditional rubber toughened polypropylene.^13,14^ POE is a thermoplastic elastomer with a relatively narrow molecular weight distribution and uniform short branched chain distribution synthesized with metallocene as a catalyst. Compared with rubber toughened PP, POE toughened PP has better processability, and its weatherability and low temperature impact resistance are improved.^15^ As a new generation of elastomer, ethylene-octene block copolymer (OBC) has better mechanical and thermal properties than traditional elastomers such as POE.^16^ The special block molecular structure of OBC gives it properties that many other elastomers do not possess. Compared with other elastomers (POE, Styrene–ethylene–butylene–stryrene, *etc.*), OBC has better impact resistance, better compression deformation resistance and higher heat resistance temperature.^17^ Pardo *et al.*^18^ toughened PP/OBC blends with ash and they found that the fracture absorption energy of the blends increased with the increase of ash content, indicating that the toughness of the blends was enhanced. Liu *et al.*^19^ studied the effect of OBC with different octene content on toughening PP. The results showed that when the octene content in soft segment of OBC was higher, the toughening effect of OBC on PP was better. Li *et al.*^20^ prepared PP/OBC nanofibers with OBC as matrix and PP as disperse fibers. The results showed that the nanofibers had excellent mechanical properties. He *et al.* added OBC in PP/HDPE blends and significantly improved the impact resistance of PP/HDPE blends.^21^

UHMWPE is a thermoplastic with excellent properties, the molecular weight of which is over 1.5 million. Compared with HDPE, UHMWPE has fewer branched chains and higher molecular weight. UHMWPE has almost all the advantages of engineering plastics such as excellent rigidity and toughness, wear resistance, corrosion resistance, self-lubrication, non-toxic and can be used as environmental protection materials. Improving the mechanical properties of PP by UHMWPE has been a research hotspot in the scientific circles. Liu *et al.*^22^ found that adding PP was more helpful to improve the fluidity of UHMWPE than adding HDPE. PP mainly concentrated on the surface of the blend and contacted the barrel of the extruder, increased the friction between the blend and the inner wall of the barrel, and improved the forward conveying ability of the material.^23^ However, on the one hand, because the molecular chains of UHMWPE are closely intertwined, so its processing is difficult resulting from the high viscosity and poor fluidity; on the other hand, because the compatibility between UHMWPE and PP is poor, purpose of improving the mechanical properties of PP by blending is not achieved. Using compatibilizers to blend with PP and UHMWPE can not only overcome the shortcomings of UHMWPE processing difficulties, but also improve the interfacial compatibility of UHMWPE and PP, making it possible for UHMWPE to strengthen and toughen PP.

In the present work, UHMWPE was used to strengthen and toughen PP, and elastomer OBC as the compatibilizers. The mechanical, thermal and rheological properties of PP/OBC/UHMWPE blends were studied by adding UHMWPE and OBC. The effect of the cross-section of the blend was observed and analyzed. Finally, the dispersion of UHMWPE in the blend was further improved by ultrasonic wave, and the mechanical properties of the blend were improved.

## 2. Experiment

### 2.1 Materials

Polypropylene (PP, PPH-T03) with the melt flow rate (MFR) of 3.6 g/10 min (230 °C, 2.16 kg) and the weight-average molecular weight (*M*_w_) of 4.1 × 10^2^ kg mol^−1^ was purchased from Sinopec Zhenhai Refining and Chemical Company Ltd. (Ningbo, Zhejiang, China). Olefin block copolymers (OBC, 9100, 12 mol% octene) with MFR of 1 g/10 min (190 °C, 2.16 kg), *M*_w_ of 160 kg mol^−1^, and the polymer dispersity index (PDI) of 2.4 were purchased from the Dow Chemical Company. Ultra-high molecular weight polyethylene (UHMWPE, 0.943 g cm^−3^) with the weight-average molecular weight (*M*_w_) of 1.5 × 10^3^ kg mol^−1^ was purchased from Sinopec Qilu Petrochemical Branch Company Ltd. (Zibo, Shandong, China). The antioxidant Irganox 1010 (pentaerythritol tetrakis-3-(3,5-di-*tert*-butyl-4-hydroxyphenyl)propionate) was purchased from the BASF (Ludwigshafen, Germany).

### 2.2 Preparation of blends

PP, UHMWPE and OBC were vacuum dried at 75 °C for 4 hours to remove the adsorbed moisture. Then they were premixed according to a certain mass ratio (Table 1), followed by adding 0.2 wt% Irganox 1010. After homogeneous mixing, they were extruded and granulated by an ultrasonic twin-screw extruder (see Fig. S.1†). The SHJ-20 co-rotating intermeshing twin-screw extruder with the screw diameter of 21.7 mm and the length to diameter ratio of 40 was produced by the Nanjing Jieya Company. The extruder was divided into eight segments and each segment was composed of a separated heating and water-cooling system. The temperature of the first to the seventh segments of the extruder is 170 °C, 180 °C, 190 °C, 200 °C, 200 °C, 195 °C, 190 °C, the temperature of the head is 190 °C, the feed rate was maintained at 10 rpm with the screw speed of 200 rpm and the flow rate of the die of about 7.4 g min^−1^. We installed two ultrasonic launchers produced by the Shanghai Shengxi Instrument Company (China) at the head of the extruder with the frequency of 20 kHz and a power range of 0 to 600 W. The diameter of the ultrasonic horn tips was 16 mm, and the gap in the ultrasonic region was 8 mm with the width of 13 mm in the elliptical channel.

**Table 1:** Preparation parameters for PP/OBC/UHMWPE blends^a^

Sample	PP (g)	OBC (g)	UHMWPE (g)	Ultrasonic power (W)
PP	100	—	—	—
P/U(99/1)	99	—	1	—
P/O/U(99/3/1)	99	3	1	—
P/O/U(99/6/1)	99	6	1	—
P/O(99/6)	99	6	—	—
P/O/U(99/6/0.5)	99	6	0.5	—
P/O/U(99/6/1.5)	99	6	1.5	—
P/O/U(99/6/2.0)	99	6	2.0	—
P/O/U(99/6/1.5)-200	99	6	1.5	200
P/O/U(99/6/1.5)-400	99	6	1.5	400
P/O/U(99/6/1.5)-600	99	6	1.5	600

a The ultrasonic power is the power of one launcher.

After drying, the performance was tested.

### 2.3 Mechanical properties

Tensile test was carried out according to the GB/T16421-1996 test method using an Instron 3367 test machine with a cross-head speed of 50 mm min^−1^. Dumbbell-type samples was cut with a thickness of 2 mm and length of 20 mm from the tablet samples for the tensile test. The tablet samples were pressed in a tableting machine (Model GT-7014-P, GOTECH Instrument Company, Taiwan) through the following process. Firstly, dried pellets were placed in the mold and preheated for 5 min at 200 °C, and the samples were pressed at 8 MPa for 5 min after 15 exhaust circulations at 5 MPa. Then the tablet samples were cooled by water circulation for 3 min.

The impact strength of injection-molded samples was measured according to the GB/T1843-2008 test method using an Izod impact test machine (Model 9050, CEAST Company, Pianezza, Italy). Injection-molded samples were prepared in a micro-injection molding machine (Model Minijet, HAAKE Instrument Company, Germany) through the following process. The dried pellets were placed in a cylinder and preheated for 5 min at 200 °C. Then samples were injected into the mold and maintained at 90 °C with the injection pressure of 1000 bar and the pressure keeping time of 20 s. We obtained an impact spline with a thickness of 4 mm, width of 10 mm, and notch-bottom radius of 0.25 mm after cooling the mold at room temperature.

### 2.4 Thermal measurements

Differential scanning calorimeter (Model DSC Q200, TA Instrument Company, New Castle, DE, USA) was used to test the thermal performance of the prepared samples. Firstly, 5 mg samples were placed in the testing chamber and raised the temperature to 200 °C at 10 °C min^−1^. The temperature of the test chamber was kept at 200 °C for 5 min to eliminate the thermal history. Then the temperature was decreased from 200 °C to 40 °C at 10 °C min^−1^ and the crystallization curves were recorded. Next the temperature was raised to 200 °C at 10 °C min^−1^ and the melting curves were recorded. The whole process was performed under a nitrogen atmosphere with a flow rate of 50 mL min^−1^.

The formula for calculating the crystallinity of samples is as follows:1
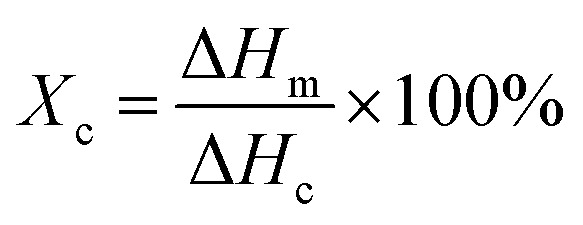
where Δ*H*_m_ represents the melting enthalpy (J g^−1^) of the sample, Δ*H*_c_ represents the melting enthalpy of 100% crystalline PP or PE; Δ*H*_c_ PP is 209.2 J g^−1^ (ref. 24) and Δ*H*_c_ PE is 290 J g^−1^.^25^

### 2.5 Morphological characterization

The morphologies of the prepared samples were characterized using a field emission scanning electron microscope (FE-SEM) produced by the FEI Company (Model NOVA Nano SEM450, Hillsboro, OR, USA). The tablet samples were first fractured in liquid nitrogen, and then the fractured cross-section was etched with *n*-heptane for 5 hours and covered with platinum.

### 2.6 Rheological analysis

The dynamic rheological properties of the samples were studied using the parallel-plate-mode of the TA ARES rheometer. The samples were tested with a thickness of 2 mm and diameter of 25 mm at 200 °C. We applied the fixed strain amplitude of 2% and the frequency range of 0.1 to 100 rad s^−1^.

### 2.7 Contact angle measurement

The static contact angle of the substrate were measured using the sessile drop method of the contact angle meter (Model OCA20, Datatphysics Company, Stuttgart, Germany) relative to water and diiodomethane (3 μL). Surface energy, interfacial tension and adhesive energy were calculated by the Wu's equations (eqn (1)–(5)), where *θ* is the contact angle, *γ* is the surface energy (mN m^−1^), *γ*^p^ and *γ*^d^ are the polar component and the dispersion component of surface energy respectively, indices 1 and 2 denote different materials, *γ*_12_ is the interfacial tension (mN m^−1^) between two phases, and *W*_12_ is the adhesive energy of two phases (mN m^−1^).2
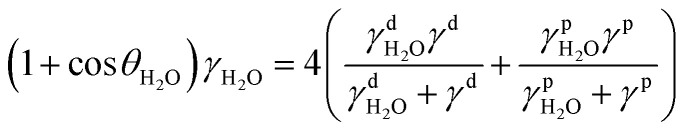
3
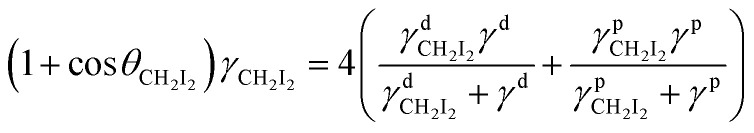

4 *γ* = *γ*^d^ + *γ*^p^

5
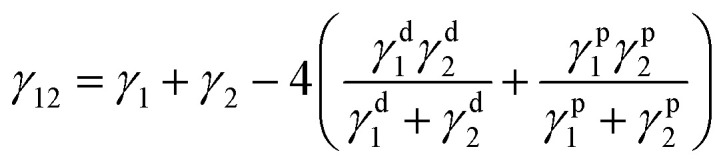

6 *W*_12_ = 2(*γ*^d^_1_*γ*^d^_2_)^1/2^ + 2(*γ*^p^_1_*γ*^p^_2_)^1/2^

## 3. Results and discussion

### 3.1 Mechanical properties of the blends

**Fig. 1:**
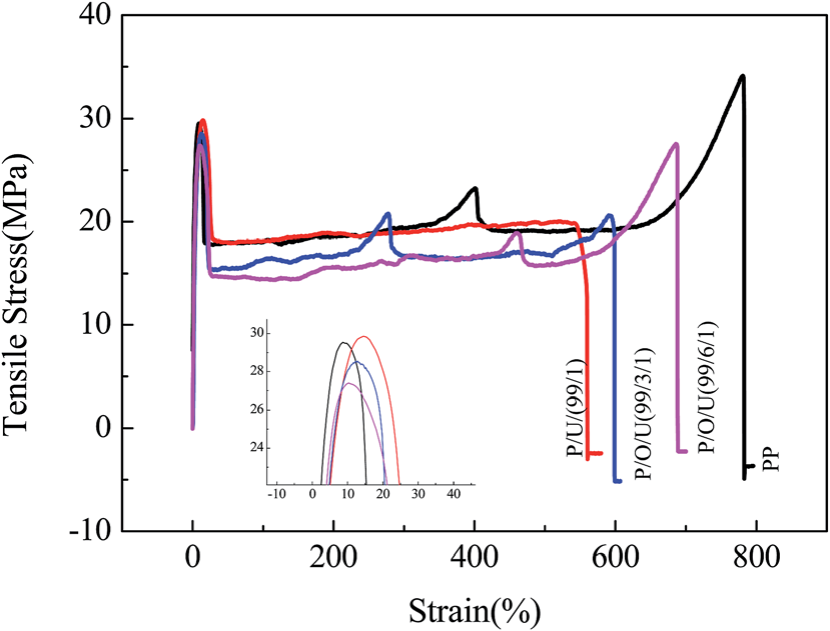
The tensile stress–strain curves of PP/OBC/UHMWPE blends with different OBC contents.

The effect of OBC content on the tensile stress–strain curve of the blends was shown in Fig. 1. It can be seen that the elongation at break and the absorption energy at break of PP/UHMWPE blends decreased obviously compared with PP. The notch impact strength data of PP/UHMWPE blends showed that the impact strength of PP/UHMWPE blends was not improved but decreased because of the poor compatibility between PP and UHMWPE. So simple blending can't toughen PP.

**Fig. 2:**
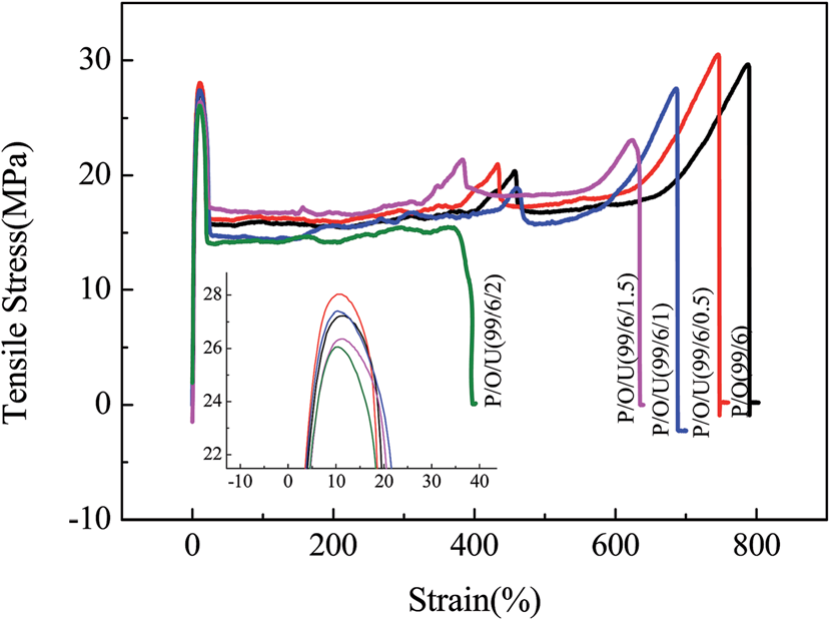
The tensile stress–strain curves of PP/OBC/UHMWPE blends with different UHMWPE contents.

Fig. 2 and Table 2 showed that with the increase of the content of OBC, the notched impact strength of PP/OBC/UHMWPE blends increased significantly, while the tensile yield strength decreased slightly. When the content of OBC was 6 wt%, the impact strength of the blends reached 10.4 kJ m^−2^ and the tensile yield strength is 27.4 MPa. Compared with pure PP, the impact strength increased by 70% and the tensile strength decreased only by 7%. On the other hand, with the increase of UHMWPE content, the tensile yield strength and impact strength of PP/OBC/UHMWPE blends increased first and then decreased. When the UHMWPE content was 1%, the mechanical properties of the blends were the best. And when the UHMWPE content exceeded 1 wt%, the impact strength and tensile yield strength of the blends began to decrease gradually. The reason was that UHMWPE, unlike HDPE, has excellent tensile properties. When a small amount of UHMWPE was added to PP/OBC blends, it can be strengthened and toughened at the same time. When the amount of UHMWPE was high, it is difficult to disperse homogeneously in the blends due to its high viscosity when blending with PP and OBC. The mechanical properties of the blends decreased gradually due to the mechanical defects of the blends.

**Table 2:** Mechanical properties of PP/OBC/UHMWPE blends

Sample	Tensile yield strength (MPa)	Elongation at break (%)	Impact strength (23 °C, kJ m^−2^)	Impact strength (0 °C, kJ m^−2^)
PP	29.5	798	6.1	2.2
P/U(99/1)	29.8	577	5.5	1.9
P/O/U(99/3/1)	28.6	608	7.8	3.1
P/O/U(99/6/1)	27.4	701	10.4	5.5
P/O(99/6)	27.0	802	8.5	4.3
P/O/U(99/6/0.5)	27.9	760	9.7	4.9
P/O/U(99/6/1.5)	26.3	635	8.5	4.2
P/O/U(99/6/2.0)	26.0	410	8.3	3.0

At the same time, when the addition of UHMWPE was less than 1.0 wt%, the notched impact strength of blends at low temperature increased gradually with the addition of UHMWPE, showing excellent impact resistance at low temperature.

Usually, PP and PE are semi-crystalline polymers, which have two parts: crystalline and amorphous regions. The molecular chains in crystalline regions are arranged regularly, and chain winding and slipping are not easy to occur. The molecular chains in non-crystalline regions are relatively free. They can form ring molecules, tethered molecules, entangled chains and other structures. The chain morphology of crystalline region has an important influence on the mechanical properties of materials. Generally speaking, the tighter the entanglement is, the more the lacing molecules are, the better the mechanical properties of materials, such as UHMWPE, which has a very long polymer chain and there are many random entanglement chains formed in the amorphous region of UHMWPE. Unwrapping requires a lot of energy, so UHMWPE has excellent mechanical properties. When UHMWPE is added to PP, due to their poor compatibility, the molecular chains of PP and UHMWPE repel each other and form fewer entanglement points. Therefore, UHMWPE acts as the stress concentration point when the blend is subjected to stress, and its contact molecules with PP are easily disentangled, which results in matrix peeling and reduces the mechanical properties of the blend. With the addition of elastomer OBC, the compatibility between PP and UHMWPE is improved. Its hard segment can form winding structure with UHMWPE, while the soft segment can form winding structure with PP. As a bridge, PP and UHMWPE are tightly bound together, which significantly increases the chain winding density of blends and makes blends more flexible.^21^ The entanglement of molecular chains consumes a lot of energy and improves the mechanical properties of the blends remarkably.

### 3.2 Section morphology of the blends

**Fig. 3:**
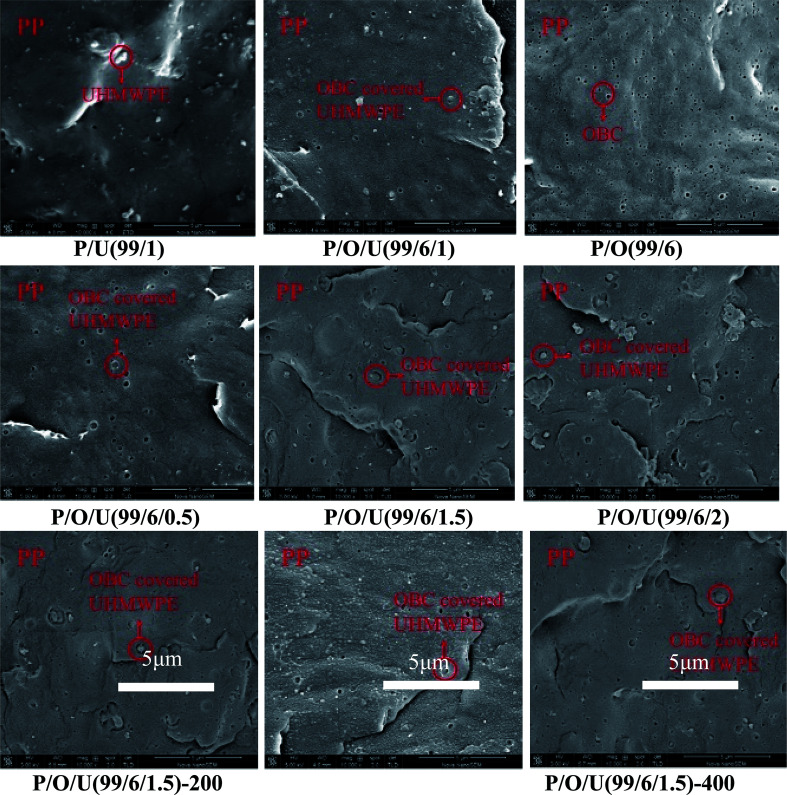
SEM images for fracture surfaces of PP/OBC/UHMWPE blends.

**Fig. 4:**
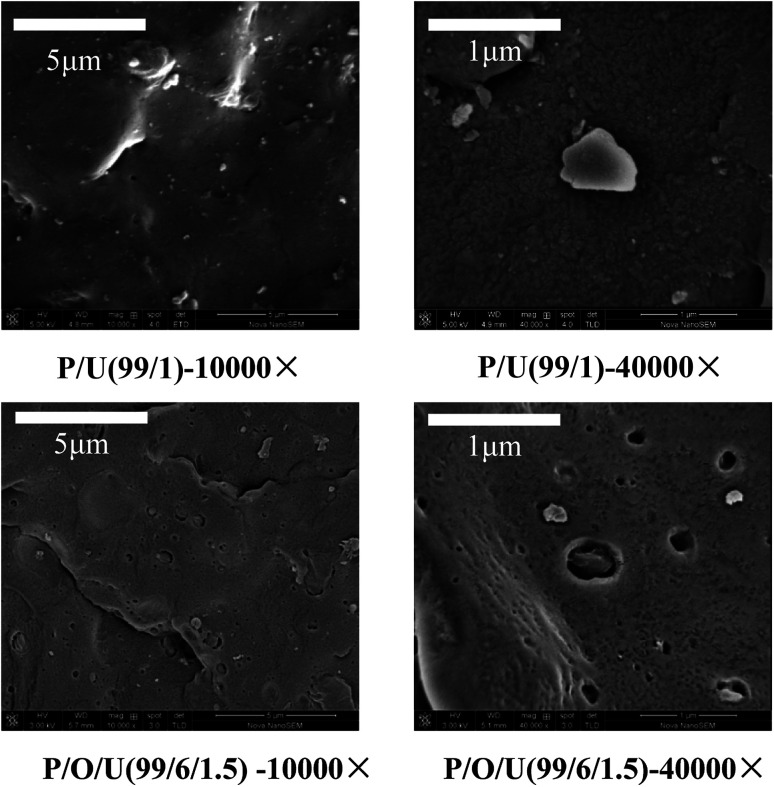
SEM images of PP/OBC/UHMWPE blends with different magnifications.

The cross sections of blends with different OBC and UHMWPE contents were scanned by SEM to analyze the dispersed morphology of PP, OBC and UHMWPE. As shown in Fig. 3, it can be seen that the PP/UHMWPE blends formed the cross section morphology with UHMWPE as the dispersed phase and PP as the continuous phase. Due to the poor fluidity of UHMWPE, it can be seen that the dispersion of UHMWPE in PP was not uniform, and phase separation occurred between UHMWPE and PP. The cross section of PP/OBC/UHMWPE showed that the blend formed a coating structure with OBC as shell and UHMWPE as core. OBC was dispersed on the interface between PP and UHMWPE. At this time, UHMWPE was embedded in PP matrix, which indicated that OBC significantly improved the compatibility between PP and UHMWPE. When the OBC content was 3 wt%, we can see that some UHMWPE particles have been encapsulated by OBC. When the OBC content reached 6 wt%, UHMWPE was almost completely encapsulated by OBC. On the other hand, when the content of UHMWPE was less than 1 wt%, all the particles were surrounded by OBC. When the content of UHMWPE exceeded 1 wt%, some UHMWPE was directly dispersed in PP matrix (Fig. 3). When the content of UHMWPE reached 2 wt%, significant UHMWPE aggregates were formed. The SEM with higher magnification was shown in the following figures (Fig. 4) of the ESI.†

### 3.3 Formation mechanism of section morphology

The contact angles of PP, OBC and UHMWPE were measured in order to analyze the dispersed forms of PP, OBC and UHMWPE in blends. As shown in Table 3 the surface energy of PP, OBC and UHMWPE was calculated by using formulas (2), (3) and (4).

**Table 3:** Surface energies of PP, OBC, and UHMWPE

Sample	Contact angle *θ*H_2_O (°)	Contact angle *θ*CH_2_I_2_ (°)	Surface energy *γ* (mN m^−1^)	Dispersion component *γ*^d^ (mN m^−1^)	Polar component *γ*^p^ (mN m^−1^)
PP	151.1	148.9	34.7	33.4	1.3
OBC	144.5	145.8	36.9	34.4	2.5
UHMWPE	100.2	121.8	36.1	34.4	1.7
H_2_O	—	—	72.8	21.8	51
CH_2_I_2_	—	—	50.8	48.5	2.3

**Fig. 5:**
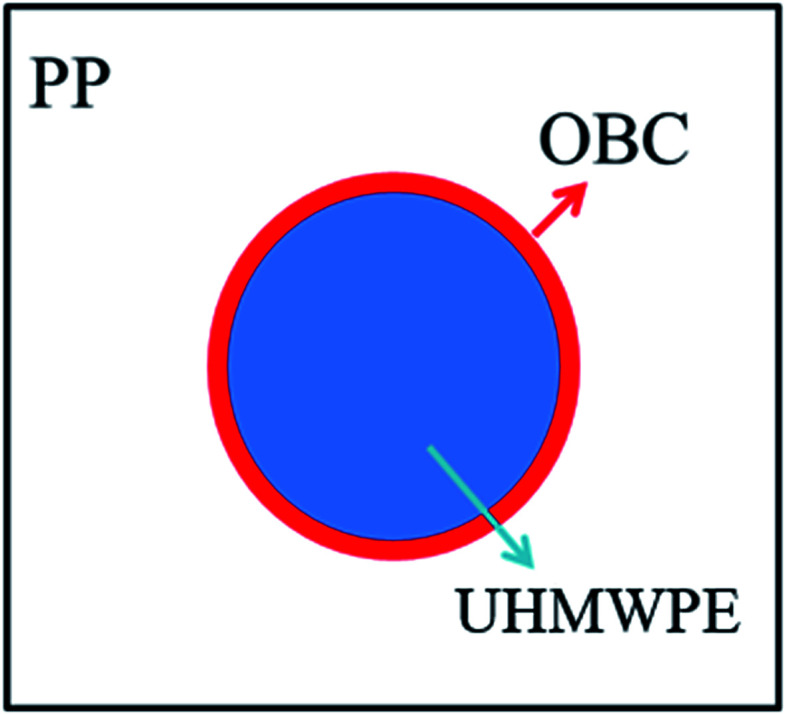
The core–shell structure of the blends.

In order to analyze the interfacial interaction between PP, OBC and UHMWPE, the interfacial tension and adhesion work of the three materials were calculated by using formulas (5) and (6), which are listed in Table 4. It can be seen that the adhesion work of UHMWPE and PP was obviously greater than that of OBC. In addition, UHMWPE has more chain winding structure, and its fluidity is lower. When blended with PP and OBC, UHMWPE tends to bind to OBC with the same viscosity. Therefore, the blends will form OBC-coated UHMWPE structure, as shown in Fig. 5.

**Table 4:** Interfacial tensions and adhesive energies among PP, OBC, and UHMWPE

Sample	Interfacial tension *γ*_12_ (mN m^−1^)	Adhesive energy *W*_12_ (mN m^−1^)
PP/OBC	0.4	71.4
OBC/UHMWPE	0.15	73.0
PP/UHMWPE	0.07	71.0

### 3.4 Thermal properties of the blends

**Fig. 6:**
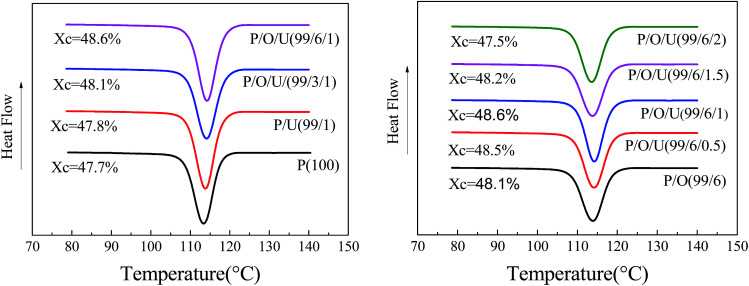
Crystallization curves of PP/OBC/UHMWPE blends with different OBC contents.

**Fig. 7:**
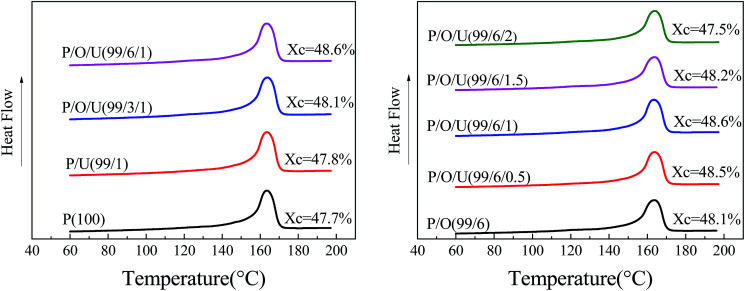
Melting curves of PP/OBC/UHMWPE blends with different UHMWPE contents.

The thermal properties of PP/OBC/UHMWPE blends were shown in Fig. 6 and 7, Table S.3.† The crystallinity of PP/OBC/UHMWPE blends increased first and then decreased with the increase of UHMWPE content. When the content of UHMWPE was 1 wt%, the crystallinity of PP/OBC blends increased from 48.1% to 48.6%, and when the content of UHMWPE exceeded 1 wt%, the crystallinity of the blends began to decrease, which indicated that the addition of UHMWPE had a competitive effect on the crystallization behavior of the blends. On the one hand, the existence of UHMWPE as a heterogeneous nucleus was conducive to improving the crystallinity of the polymers. On the other hand, the interaction between UHMWPE and polymer chains hindered the movement of macromolecular chains and the orderly arrangement of molecular chains, thus reducing the crystallinity of polymers. When the content of UHMWPE was less than 1 wt%, its dispersion in blends was more uniform, and heterogeneous nucleation of UHMWPE played a leading role. Therefore, the crystallinity of blends showed an upward trend. When the content of UHMWPE was over 1 wt%, it formed obvious aggregates, hindered the movement of molecular chains and destroyed the orderly arrangement of molecular chains. The crystallinity of the blends tended to decrease.

In addition, it can be seen that the crystallization temperature and crystallinity of the blends increased with the increase of OBC content. This is because as a compatibilizer, OBC improved the interface compatibility between PP and UHMWPE, which was more likely to induce heterogeneous nucleation at the interface.

### 3.5 Rheological properties of PP/OBC/UHMWPE blends

**Fig. 8:**
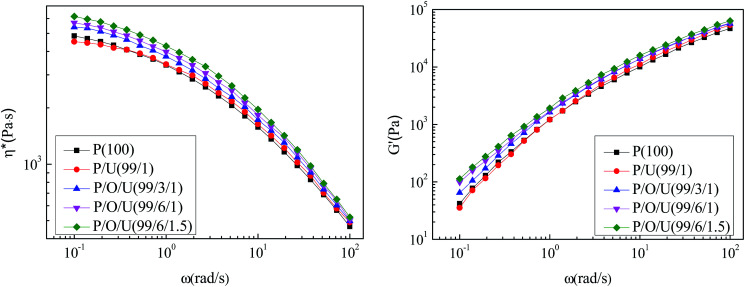
Viscosity–shear frequency (left) and storage modulus–shear frequency (right) curves of PP/OBC/UHMWPE blends.

As shown in Fig. 8 (left), the blend viscosity–shear frequency curve shows that in low frequency, the viscosity of PP/UHMWPE blends was obviously lower than that of pure PP. Because UHMWPE had higher molecular weight than PP and they had poor compatibility. The polymer chains of UHMWPE and PP were mutually exclusive. A small amount of UHMWPE dispersed in PP acts as lubricant. (Mutual exclusion decreased friction between chains) reduced the flow resistance of PP. With the addition of elastomer OBC, the viscosities of blends increased obviously. On one hand, OBC improved the compatibility of PP and UHMWPE, and increased the degree of molecular chain entanglement between them; on the other hand, OBC had higher viscosities, which would lead to the increase of the viscosities of blends. As shown in Fig. 8 (right), the energy storage modulus–shear frequency curve of blends proved that in low frequency region the addition of elastomer OBC improved the energy storage modulus of blends. The addition of OBC marked PP and UHMWPE molecular chains entangled tightly, which made the relaxation time of blends longer and the storage modulus higher in low frequency region. In general, OBC and UHMWPE had little effect on the viscosity and storage modulus of the blends, and the blends still had good processing properties.

### 3.6 Melt flow rate of PP/OBC/UHMWPE blends

The MFR of the blends was tested in order to investigate the processing fluidity of the blends. The addition of UHMWPE (1 wt%) in PP increased the MFR of the blends and improved the fluidity. The addition of OBC decreased the MFR of the blends slightly. MFR of the blends ranged from 2.8 to 3.8 g/10 min in general, proved a good fluidity.

This also gave us a revelation that although UHMWPE was difficult to process and apply, it could improve the mechanical properties of materials while have little effect on the processing properties of polymers by adding a small amount of UHMWPE. And its mechanical properties were tested and section morphology was recorded.

### 3.7 Effect of ultrasonic irradiation on properties of PP/OBC/UHMWPE blends

**Fig. 9:**
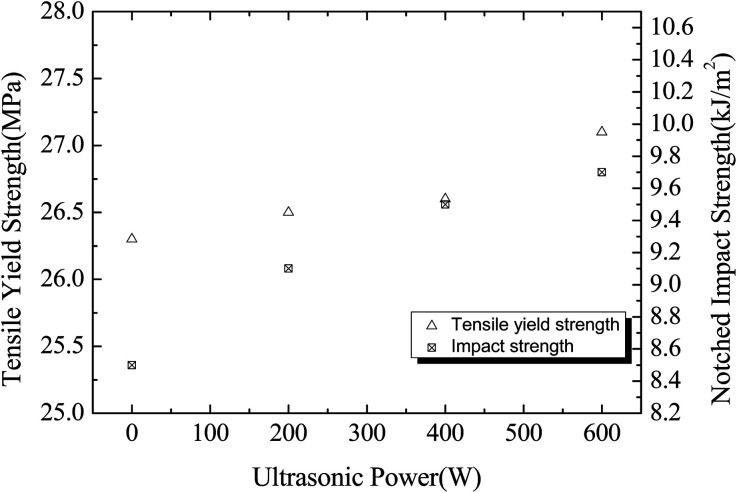
Mechanical properties of PP/OBC/UHMWPE blends at different ultrasonic power levels.

Compared with the blends without ultrasonic irradiation, UHMWPE was easy to form aggregates because of its poor fluidity. For example, phase separation between UHMWPE and PP exists in a blend PP/OBC/UHMWPE with 1.5 wt% UHMWPE. Therefore, UHMWPE was treated by ultrasound irradiation in order to improve the dispersion of UHMWPE in blends, and its mechanical properties were tested and section morphology was recorded and shown in Fig. 9. The molecular weight, molecular distribution and mechanical properties of PP were not changed under the ultrasound as shown in Tables S.1 and S.2.† It can be seen that with the increase of ultrasonic power, the tensile yield strength and impact strength of the blends increase gradually. Compared with the blends without ultrasonic irradiation, the tensile yield strength and impact strength of the blends obtained by ultrasonic 600 W increased by 0.8 MPa and 1.2 kJ m^−2^ respectively.

SEM analysis showed that the phase separation between PP and UHMWPE disappeared after ultrasonic treatment (Fig. 3). UHMWPE had smaller particle size and were almost embedded in PP matrix. This was because ultrasound improved the compatibility between PP and UHMWPE and made UHMWPE dispersed uniformly in PP matrix, and thus decreased the size of PP/OBC/UHMWPE core–shell structures,^17^ which played a role in strengthening and toughening PP.

**Fig. 10:**
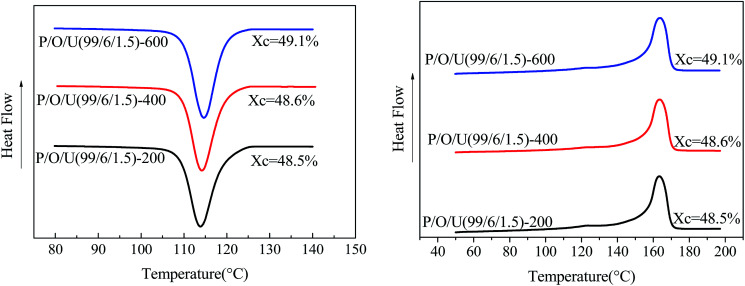
Crystallization (left) and melting (right) curves of PP/OBC/HDPE blends at different ultrasonic power levels.

The crystallization and melting curves of the blends treated with different ultrasonic power are shown in Fig. 10 and Table S.4.† It can be seen that the crystallization peak of the blends shifts to the right with the increase of ultrasonic power. Under 600 W ultrasonic power, the crystallization temperature of the blends increases by 1.2 K. This was because UHMWPE aggregates were broken by ultrasound, which made UHMWPE uniformly dispersed in PP matrix, which played a role of heterogeneous nucleation of PP and improved the crystallization temperature of blends.

## 4. Conclusions

Blends of PP, UHMWPE, and OBC were prepared using a twin-screw extruder under the application of ultrasonic irradiation. The addition of UHMWPE in PP did not improve the mechanical properties due to the poor compatibility between UHMWPE and PP. The addition of elastomer OBC improved the compatibility of PP with UHMWPE and nearly doubled the notched impact strength. In addition, the SEM images of blend sections showed that PP/UHMWPE blends exhibited phase separation, but OBC almost completely dispersed on the phase interface between PP and UHMWPE, which proves its effect as an efficient compatibility modulator. Besides, the addition of OBC and UHMWPE in PP matrix played a role of heterogeneous nucleation which causes an increase of crystallization temperature of the blends. When the content of UHMWPE was over 1 wt%, the formation of a UHMWPE agglomeration structure hindered the crystallization of the blends and made the crystallization temperature decrease. UHMWPE in PP/OBC/UHMWPE blends dispersed more evenly under the action of ultrasound, and its mechanical properties were further improved. UHMWPE played a better role in heterogeneous nucleation and increased the crystallization temperature of the blends.

## Conflicts of interest

There are no conflicts to declare.

## Supplementary Material

RA-009-C9RA01073D-s001
