# Supplementary material for: Synergistic toughening of polypropylene with ultra-high molecular weight polyethylene and elastomer-olefin block copolymers
Source: RSC Adv. 2019 Aug 2;9(41):23994–4002. doi: 10.1039/c9ra01073d (PMC9069455; doi:10.1039/c9ra01073d)
Supplement: RA-009-C9RA01073D-s001 [file RA-009-C9RA01073D-s001.pdf]

Electronic Supplementary Material (ESI) for RSC Advances.
This journal is © The Royal Society of Chemistry 2019

Support information

Synergistic toughening of polypropylene with ultra-high molecular weight polyethylene and elastomer-olefin block copolymers

Lucheng Qi[1], Lei Wu[1], He Ren[3], Hui Cheng[1], Boping Liu[2], Xuelian He*[1]

[1]Shanghai Key Laboratory of Multiphase Material Chemical Engineering, East China University of Science and Technology, Shanghai 200237, China

[2]College of Materials and Energy, South China Agricultural University, Guangzhou 510642, China

[3]Daqing Petrochemical Research Center of PetroChina, Daqing 163714, China

Corresponding Authors

Xuelian He (E-mail: hexl@ecust.edu.cn) Tel: +86-21-64253364

Author Lists:

Lucheng Qi: *lcqi_1224@foxmail.com*

Lei Wu: *edwood_vip@163.com*

He Ren: *renhe459@petrochina.com.cn*

Hui Cheng: huihyva@ecust.edu.cn

Boping Liu: *boping@scau.edu.cn*

Xuelian He: *hexl@ecust.edu.cn*

Co-first author: Lei Wu

Table S.1 The effect of ultrasonic irradiation on molecular weight and molecular weight distribution of PP

Sample	M_w(kg/mol)	PDI
PP(0W)	410	4.9
PP(400W)	390	5.0
PP(600W)	380	5.0

Table S.2 The effect of ultrasonic irradiation on mechanical properties of PP

Sample	Tensile yield strength (MPa)	Elongation at break (%)	Impact strength (23℃,kJ/m^2)
PP(0W)	29.5	798	6.1
PP(400W)	29.4	810	6.3
PP(600W)	29.5	802	6.0

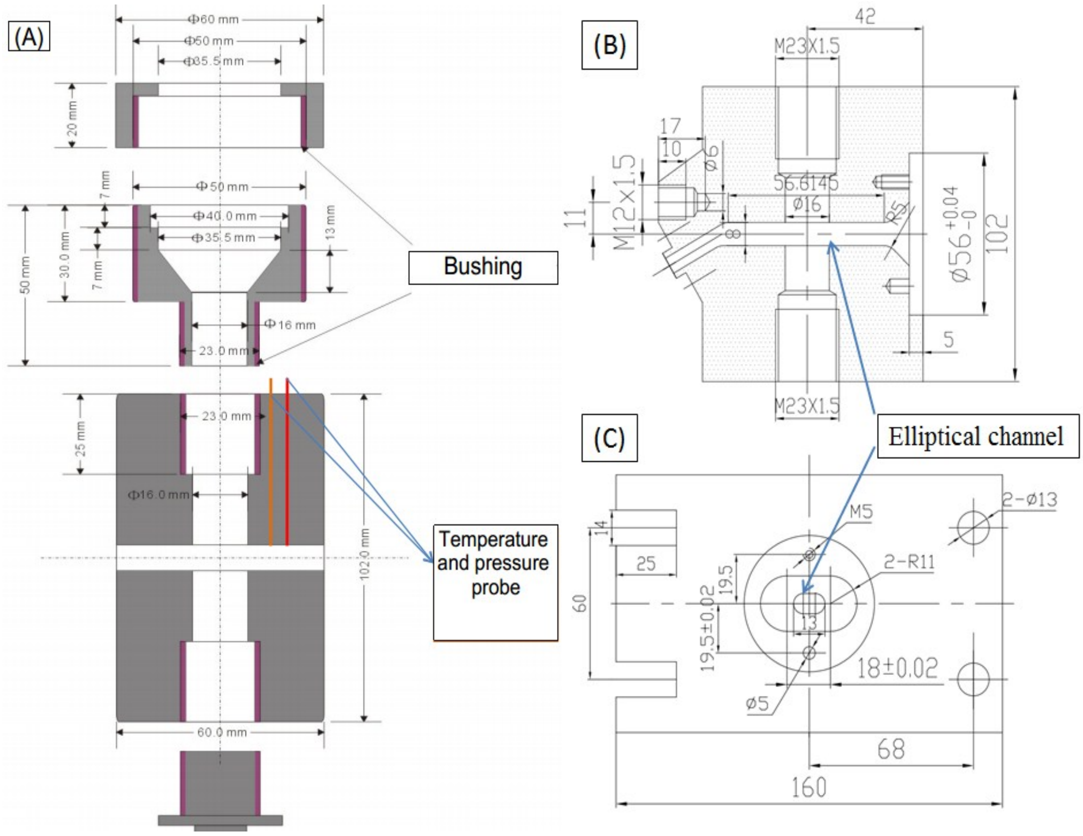

Fig. S.1 Schematics of ultrasonic reactor (A), lateral view (B) and front view of die (C).

Table S.3 The thermodynamic property data of the blends

Sample	T_m(°C)	T_c(°C)	X_c(%)
PP	163.5	113.4	47.7
P/U (99/1)	163.6	113.8	47.8
P/O/U(99/3/1)	163.6	114.1	48.1
P/O/U(99/6/1)	163.3	114.2	48.6
P/O(99/6)	163.7	113.8	48.1
P/O/U(99/6/0.5)	163.8	114.0	48.5
P/O/U(99/6/1.5)	163.8	113.7	48.2
P/O/U(99/6/2.0)	163.4	113.5	47.5

Table S.4 The thermodynamic property data of the blends treated by different ultrasonic power

Sample	T_m(℃)	T_c(℃)	X_c(℃)
P/O/U(99/6/1.5)	163.8	113.7	48.2
P/O/U(99/6/1.5)-200	163.9	113.9	48.5
P/O/U(99/6/1.5)-400	163.8	114.2	48.6
P/O/U(99/6/1.5)-600	163.9	114.6	49.1
